# Validity and Reliability of the Parental Health Literacy Questionnaire for Caregivers of Children Aged 0 to 3 Years in China

**DOI:** 10.3390/ijerph192316076

**Published:** 2022-12-01

**Authors:** Anxin Yin, Guannan Bai, Hong Jiang, Xia Xiao, Xinwen Zhang, Huaiting Gu, Min Zheng, Mu Li

**Affiliations:** 1 School of Public Health, Key Lab of Health Technology Assessment, National Health Commission of the People’s Republic of China, Fudan University, Shanghai 200032, China; 2 Department of Child Health Care, The Children’s Hospital, and National Clinical Research Center for Child Health, Zhejiang University School of Medicine, Hangzhou 310052, China; 3 School of Public Health, Kunming Medical University, Kunming 650500, China; 4 Xi’an People’s Hospital, Xi’an 710004, China; 5 School of Public Health, Jining Medical University, Jining 272067, China; 6 Yunnan Maternal and Child Health Care Hospital, Kunming 650051, China; 7 School of Public Health, The University of Sydney, Sydney, NSW 2006, Australia

**Keywords:** early childhood, caregivers, health literacy, anticipatory guidance, reliability, validity, physical health, psychological health

## Abstract

Caregivers’ health literacy plays a vital role in the quality of parenting and significantly impacts children’s physical and psychological health. However, the instruments to assess the health literacy of caregivers of children aged 0 to 3 years are lacking. This study aimed to evaluate the reliability and validity of the Chinese Parental Health Literacy Questionnaire (CPHLQ) in China. We conducted a cross-sectional study. Six hundred and thirty-four caregivers of children aged 0 to 3 years were recruited from Shandong, Yunnan, and Shaanxi Provinces, representing the eastern, central, and western regions of China, between November 2020 and January 2021. The reliability was evaluated by internal consistency reliability and split-half reliability. The construct validity was determined by confirmatory factor analysis. Social determinants of parental health literacy were assessed by multivariate linear regression model. Results showed that CPHLQ had satisfactory reliability and acceptable construct validity. Mothers compared to other types of caregivers, higher education levels, and nuclear or extended families compared to other family compositions were significantly associated with higher parental health literacy. The study further demonstrated that CPHLQ is a reliable and valid instrument to measure the health literacy of caregivers of children aged 0 to 3 years in the Chinese population. It can be used as an evaluation tool for intervention research, to inform policy-making and future health education interventions of improving caregivers’ health literacy.

## 1. Introduction

Early life development creates the foundation of child survival and holistic human development [1]. The first three years of life have been widely confirmed as a critical stage for children’s physical growth and neurodevelopment [2,3,4,5]. Appropriate parenting is essential for children’s full development [6]. Poor parenting behavior in the first three years is associated with children’s socioeconomic deprivation [7,8], limited academic performance [3], diminished physical and psychological development [9], and loss of human potential [2] in their later life.

The World Health Organization Regional Office for Europe (WHO EU) defined health literacy as “competencies to access, understand, appraise and apply health information to make decisions in everyday life concerning health care, disease prevention, and health promotion” [10]. Parental health literacy is an underlying determinant of parenting skills and behaviors as well as family nurturing environment, and can further exert a profound influence on children’s long-term health outcomes [11,12,13,14,15]. Parents with low health literacy more often had adverse parenting behaviors such as less accessibility of care [16], poorer understanding of health-related instructions or immunization schedules [12,17], errors in dosing of prescribed child medication [17], non-adherence to medication or recommendations [18], worse management of children with chronic diseases [11,15,19], and ineffective injury prevention [5,17], thus leading to poor child health outcomes including higher obesity rates [20], poorer oral health status [17,21], worse asthma outcomes [11], and more emergency department visits and hospitalizations [15,22]. In contrast, a high level of parental health literacy is related to high-quality parenting skills in early childhood, which is associated with children’s better physical growth and neurodevelopment, such as better academic performance in the long term [3,4,5]. 

Measuring parental health literacy is essential to understanding the level of parents’ knowledge, skills, and attitudes towards child nurturing and informing appropriate and personalized advice or interventions. However, there is a scarcity of reliable and valid instruments to measure parental health literacy in China or internationally. The most commonly used scales assessing health literacy, Rapid Estimate of Adult Literacy in Medicine (REALM) [23,24], Test of Functional Health Literacy in Adults (TOFHLA) [25,26], and Newest Vital Sign (NVS) [27,28,29] are for the general adult population, not specifically targeted at child caregivers [18,30,31]. The scales for measuring parental health literacy, such as Parental Health Literacy Activities Test (PHLAT) [32,33] and Parenting Plus Skills Index (PPSI) [34], were not developed specifically for young children aged 0 to 3 years. The Parental Health Literacy Activities Test (PHLAT) developed by American researchers Kumar et al. in 2010, focuses on parents’ ability to understand and apply common information about child health for caregivers of infants aged 0 to 13 months [35]. The Parenting Plus Skills Index (PPSI), developed by Australian researchers Ayre et al. in 2020, is a validated instrument for assessing functional, communicative, and critical health literacy skills of parents with children under 15 years old in the Australian context [34]. In China, scales designed specifically for child caregivers are scarce. Some studies used adult health literacy scales such as the Chinese Resident’s Health Literacy Questionnaire for evaluating parental health literacy [36,37,38,39,40]. In addition, some other instruments assessing parental health literacy usually aim at specific health conditions such as obesity [41], oral health problems [42,43], liver function injuries [44], and infectious diseases [45,46]. Currently, there is no available instrument to measure parental health literacy on comprehensive parenting skills for caregivers of young children at early life stages.

We developed an instrument to measure parental health literacy for caregivers of children aged 0 to 3 years: the Chinese Parental Health Literacy Questionnaire (CPHLQ) [47]. The conceptual framework of developing CPHLQ closely followed the definition of health literacy by the WHO EU, which aimed to measure health literacy from a multi-dimensional perspective [10,48]. We have previously assessed the reliability and validity in Shanghai [47,49]. The present study aimed to further evaluate the reliability and validity of CPHLQ in three provinces across different regions of China. 

## 2. Materials and Methods

### 2.1. Study Setting

We purposely selected three cities, including Jining City in Shandong Province, Kunming City in Yunnan Province, and Xi’an City in Shaanxi Province to represent eastern, central, and western China. In each city, we selected five research sites: one municipal hospital and four Community Health Centers (CHCs). A cross-sectional online survey was conducted at these three locations from November 2020 to January 2021.

### 2.2. Participants and Data Collection

We recruited caregivers (including mothers, fathers, grandparents, and other caregivers) of children aged 0 to 3 years who had an education level above grade three of primary school and the ability to communicate verbally or literally with our investigators. Those who were unwilling to participate or had severe mental illness were excluded from the study.

The research has obtained the ethical approval of School of Public Health, Fudan University (IRB# 2020-04-0820). The study was conducted in accordance with the Declaration of Helsinki [50], and followed the anonymous and voluntary principles. Child health care doctors in our selected sites were invited and trained in recruitment and data collection following a standardized protocol. During the clinic, the trained child care doctors invited eligible child caregivers to join the survey and provided participants with the self-administered questionnaire via the online WeChat platform. The online questionnaire includes four parts: social-economic characteristics of the child and family, parental health literacy on children’s physical development (CPHLQ-physical part), parental health literacy on children’s psychological development (CPHLQ-psychological part), and child’s health status. Before the survey, a brief introduction, including the aim and contents of the online questionnaire and the estimated time of completion, was provided online. Caregivers could decide whether to continue and participate in the survey. Informed consent was obtained from every participant. For those who completed the whole questionnaire, 10 RMB (approximately 1.5 USD) was provided as a reward.

### 2.3. Sampling Methods

The study used a multi-stage stratified sampling method. Based on previous studies, a sample size of minimum of 500 participants for a scale validation is considered very good [51]. The subject-item ratio is also frequently used to determine the required sample size and is recommended to be at least 5–10 for good study validation [52]. Considering the number of items in the scale and the feasibility, we planned to recruit at least 200 caregivers from each city, 600 participants in total. In each of the three cities, we recruited 50 participants from the municipal hospital and 150 participants were recruited from four Community Health Centers (CHCs). For the CHCs sampling, we first randomly selected one urban district and one suburban district from each city. Then, in each district, we recruited 75 caregivers. The 75 caregivers were recruited from two selected communities representing high and low social-economic status, using a proportional sampling approach, according to the number of children aged 0–3 in each community. Child health care doctors of study sites ensured an equal proportional sampling of children ≤ 1, 1–2, and 2–3 years old. In total, 678 caregivers gave consent and completed the survey online. After excluding 44 incomplete questionnaires, 634 completed questionnaires were included in the data analysis. Among them, 203 were from Jining, 220 from Kunming, and 211 from Xi’an.

### 2.4. Questionnaires and Measurements

#### 2.4.1. Chinese Parental Health Literacy Questionnaire (CPHLQ)

The Chinese Parental Health Literacy Questionnaire (CPHLQ) developed by the School of Public Health of Fudan University [47,49] consists of 39 items in the physical development part and 35 items in the psychological development part, thus, 74 items in total. For both physical and psychological parts, there are three domains: health care (HC), disease prevention (DP), and health promotion (HP). Each domain has four information processing factors (accessing, understanding, appraising, and applying) [47].

#### 2.4.2. Socio-Demographic Characteristics

During the survey, we collected the following participants’ socio-demographic information: the relationship between caregivers and children, permanent residence registration of children, age and sex of children, only child or not, marital status of parents, educational level of caregivers, and family composition.

#### 2.4.3. Scoring Rules

The CPHLQ contains three question types: single-choice questions, multiple-choice questions, and 4-point Likert scales. For single-choice questions, choosing the correct option will get 4 points, and choosing the wrong option will get 0 points. For multiple-choice questions containing n options, making the correct judgment of an option will get 4/n points. The total score of the multiple-choice question is the sum of each option’s points. For 4-point Likert scales, scores range from 1 (“very difficult”) to 4 (“very easy”). There is another option “don’t know” for all questions which is assigned with 0 points. When calculating the scores, the total scores of the physical and the psychological parts of CPHLQ were transformed into the centesimal system. The full score of each question was determined by its weight based on the results of Delphi expert consultation [47]. The total score of CPHLQ was the sum of the points of the physical part and the psychological part, thus ranging from 0 to 200.

### 2.5. Data Analysis

#### 2.5.1. Reliability Analysis

The online questionnaire results were exported and sorted in Excel, and analyzed by SPSS 25.0 (IBM Corporation, Chicago, USA). Based on the reliability and validity evaluation method of the Health Literacy Scale published by the WHO EU [48], the analysis of the physical scale and psychological scales were analyzed for three subscales. Reliability analysis included internal consistency reliability and split-half reliability. We used Cronbach’s α coefficient to evaluate the internal consistency reliability; the Spearman-Brown coefficient to evaluate the split-half reliability between odd questions and even questions. A reliability coefficient above 0.7 was acceptable for the whole scale, and a coefficient above 0.9 indicated excellent reliability. The subscale reliability coefficient above 0.6 was considered acceptable, above 0.7 as good, and above 0.8 as excellent [53].

#### 2.5.2. Validity Analysis

Validity was analyzed using content validity and construct validity. AMOS 23.0 was used to analyze the construct validity. The construct validity of the questionnaire was evaluated by confirmatory factor analysis. The physical scale and the psychological scale were both divided into three subscales: health care (HC), disease prevention (DP), and health promotion (HP). Confirmatory factor analysis was conducted for each of the three health literacy domains. Items were loaded onto four factors related to accessing, understanding, appraising, and applying health information. The good model fit was defined as: (1) relative chi-square (χ^2^/df) <5; (2) root mean square error of approximation (RMSEA) <0.08; (3) goodness-of-fit index (GFI) >0.9; (4) comparative fit index (CFI) and incremental fit index (IFI) >0.9; (5) parsimonious normed fit index (PNFI) >0.5 [54].

#### 2.5.3. Floor and Ceiling Effect

To test the scale reliability and validity of CPHLQ, we analyzed the floor and ceiling effect of the questionnaire [55]. A percentage of 15% or more participants obtaining the minimum or maximum score was considered a significant floor or ceiling effect, respectively, which would reduce the discriminating power of CPHLQ [56].

#### 2.5.4. Statistical Analysis

To study the influencing factors of parental health literacy, we used the independent t-test and one-way ANOVA to explore the relationship between each factor and the parental health literacy scores. To determine the practical contribution of the factors, we calculated Cohen’s *d* for independent t-tests and partial eta squared ($\eta_{p}^{2}$) for ANOVAs to estimate the effect size [57]. Based on benchmarks suggested by Cohen [58], effect sizes were indicated as small (*d* = 0.2, $\eta_{p}^{2}$ = 0.01), medium (*d* = 0.5, $\eta_{p}^{2}$ = 0.06), and large (*d* = 0.8, $\eta_{p}^{2}$ = 0.14) effects by Cohen’s *d* and $\eta_{p}^{2}$ [59]. We used multivariate linear regression to assess the social determinants associated with parental health literacy. The above data analyses were computed by using SPSS software (version 25.0, IBM Corporation), AMOS software (version 23.0, IBM Corporation), and JASP software (version 0.16.2.0). The significance level was set as *p* < 0.05 [60].

## 3. Results

### 3.1. Participants’ Characteristics

The characteristics of caregivers and their children are shown in Table 1. Among the 634 caregivers, 490 (77.29%) were mothers, and 144 (22.71%) were other caregivers, including fathers, grandparents, relatives, and other child caregivers. Meanwhile, 561 (88.49%) parents had a senior high school or above educational level. For children’s characteristics, 524 (82.65%) children in the survey had a local permanent residence registration. The boys:girls ratio was 1.15:1. The age distribution showed that 237 (37.38%) children were younger than 1 year old; 199 (31.39%) children were aged between 1 and 2 years old; 198 (31.23%) were between 2 and 3 years old.

### 3.2. Reliability

For the CPHLQ-Physical part, the Cronbach’s α coefficient of the total questionnaire was 0.915, and the Spearman-Brown coefficient was 0.889, indicating satisfactory internal consistency reliability and split-half reliability. The Cronbach’s α coefficients of the three physical subscales were 0.619 to 0.874, and the Spearman-Brown coefficients were 0.650 to 0.847, which were all acceptable. The specific coefficients are shown in Table 2.

For the CPHLQ-Psychological part, the Cronbach’s α coefficient of the total questionnaire was 0.918, and the Spearman-Brown coefficient was 0.870. The reliability coefficients of the psychological questionnaire were all >0.8. The reliability coefficients of the three psychological subscales were >0.7, indicating that they all had good reliability. The specific coefficients are shown in Table 3.

### 3.3. Construct Validity

The construct validity was evaluated by confirmatory factor analysis using the maximum likelihood method. The accessing, understanding, appraising, and applying of each subscale were used as latent variables to construct the theoretical framework. For both the physical and psychological parts of CPHLQ, the three models’ relative chi-squares were less than 5, and the other indicators also met the criteria. The results of the physical and psychological questionnaire models are shown in Table 4 and Table 5.

### 3.4. Floor and Ceiling Effect

The average score of caregivers was 154.42 ± 24.57, with the total score of CPHLQ ranging from 0 to 200. Caregivers’ final scores ranged from 35.61 to 193.22, no floor or ceiling effect was identified in the total scale. The floor and ceiling effects of the physical and psychological parts of CPHLQ and three subscales (HC, DP, HP) are shown in Table 6. The percentage of people with the lowest scores or the highest scores in subscales ranged from 0.00% to 0.01% in both the physical part and the psychological part. No significant floor or ceiling effects were found as percentages of scores at the floor or ceiling in all subscales were less than 15%. Therefore, CPHLQ can measure the health literacy of child caregivers and distinguish between high-literacy and low-literacy caregivers, which is an indicator of its satisfactory reliability and validity [55].

### 3.5. Social Determinants of Parental Health Literacy

Table 1 presents the results of the independent t-test, ANOVA, and effect size evaluation. Mothers had higher parental health literacy scores than fathers or other respondents (Cohen’*d* = 0.387, t = 3.799, *p* < 0.001). Caregivers with high education levels had higher parental health literacy scores than caregivers with low education levels of junior high school or below ($\eta_{p}^{2}$ = 0.045, F = 14.78, *p* < 0.001). The family composition had the highest effect size (Cohen’ *d* = 1.148). Table 7 presents the results of multivariate linear regression analysis. Respondents as mothers compared to fathers or other respondents (β = 0.152, 95% CI: 0.076–0.228, *p* < 0.001), high education levels compared to junior high school or below (β = 0.201, 95% CI: 0.077–0.325, *p* = 0.002 and β = 0.322, 95% CI: 0.197–0.446, *p* < 0.001), and nuclear or extended family compared to single-parent family or others (β = 0.128, 95% CI: 0.047–0.209, *p* = 0.002) were significantly associated with higher parental health literacy scores.

## 4. Discussion

Based on the health literacy theoretical model proposed by the WHO EU, we developed an instrument, CPHLQ, to measure the health literacy of caregivers of children aged 0 to 3 years [47], with health care, disease prevention, and health promotion as the first-level indicators and acquiring, understanding, appraising, and applying as the second-level indicators. In the present study, we conducted a cross-sectional study to evaluate its psychometric properties among caregivers in the eastern, central, and western areas of China. The CPHLQ was proved to have satisfactory reliability and validity, which can be applied nationwide in China to measure the parental health literacy of caregivers with children aged 0 to 3 years.

In this study, we included both the physical and psychological parts in the CPHLQ [49] and evaluated the validity and reliability of the two parts of CPHLQ separately. All coefficients met the established criteria widely used in other studies [53,54,61,62,63,64]. The content validity was not included in this study as the details have been described in our previous study [47]. This study provides further evidence that the CPHLQ has relatively good reliability and validity; it can be used to assess the parental health literacy of caregivers with children aged 0 to 3 years across China. Further, we evaluated the validity and reliability of the CPHLQ in participants from eastern, central, and western China, indicating a wider application of the scale. The instrument filled the gap of comprehensive health literacy assessment for caregivers of 0- to 3-year-old children [47].

Consistent with other studies in China [37,65], mothers achieved significantly higher CPHLQ scores than other types of caregivers. This might be attributed to the fact that mothers are usually the main caregivers who pay more attention to parenting skills [37]. A higher education level was also significantly associated with higher parental health literacy. It can be explained that caregivers with high education have a high capacity for understanding and applying information and be more likely to actively seek relevant information from reliable sources [40]. Consistent findings have been reported by Liu et al. and Zhang et al. [37,65]. Similar to the study by Zeng et al. [66] and Ma et al. [67], compared with single-parent families or other family compositions, we found nuclear or extended family compositions had higher parental health literacy. One of the explanations could be that compared with nuclear family or extended family, families with single parent or others might have a worse growth environment for children, with more family conflicts [67], insufficient primary care and attention to children, untimely response to cues of diseases, and inadequate prevention of injuries [66]. The family composition also has the highest Cohen’s *d* effect size, indicating that single-parent families or other family compositions, other than nuclear or extended families, need more support and provision of parenting skills instruction by health care staff.

The CPHLQ evaluated in this study can be used to evaluate parental health literacy status among caregivers of children aged 0 to 3 years, thus it can help to develop targeted health education interventions. In addition, compared with other instruments focusing on a limited aspect of children’s health, such as obesity [41] and oral health problems [42,43]. The CPHLQ can serve as a comprehensive assessment tool to help recognize inadequate caregivers’ health literacy in a wide range of child health and development problems.

The CPHLQ is the first scale specifically for measuring parental health literacy of caregivers with children aged 0 to 3 years [47]. The strength of this study is that we further evaluated the CPHLQ among caregivers in different geographical areas of China. The reliability and validity results demonstrated by our study suggest that the instrument can be used for parental health literacy evaluation in China and potentially for other countries in the world. Second, our study included both physical and psychological parts of CPHLQ, providing more comprehensive evaluation findings. Third, the instrument could be used as a valid measurement, help to tailor the intervention contents and further improve caregivers’ parenting skills, behaviors, family nurturing environment and children’s early development. 

There are several limitations of this research. First, since the survey was conducted online and anonymously, we did not evaluate the test-retest reliability. Second, we excluded those with educational levels below grade three of primary school or with severe mental health issues who might not understand and complete the online questionnaire independently. Therefore, the generalizability of the study findings among caregivers with low educational levels might be limited. Further studies need to include those with low literacy or educational levels. Moreover, the interpretation of the association of marital status, family composition and parenting literacy should be cautious since the numbers of the reference group were small.

## 5. Conclusions

The present study demonstrated that the Chinese Parental Health Literacy Questionnaire (CPHLQ) is a reliable and valid instrument to measure the health literacy of caregivers of children aged 0 to 3 years in China. Further evaluation research is recommended to apply CPHLQ to an extended population. The CPHLQ can be used as a valid evaluation tool for assessing parental health literacy and tailoring intervention contents to improve caregivers’ health literacy, and potentially promote children’s health and early development.

## Figures and Tables

**Table 1:** General characteristics of caregivers and their children (N = 634).

Characteristic	N = 634		Total Score	t or F	*p*	Effect Size ^a^
	n	%	Mean ± SD			
Respondent						
Mother	490	77.29	156.74 ± 22.30	3.799	<0.001 *	0.387
Father/Other	144	22.71	146.54 ± 29.86			
Permanent residence registration						
Local residence	524	82.65	154.65 ± 24.47	0.521	0.603	0.055
Non-local residence	110	17.35	153.31 ± 25.12			
Age of children						
0–1 year	237	37.38	153.44 ± 27.14	0.312	0.732	0.001
1–2 years	199	31.39	155.20 ± 24.24			
2–3 years	198	31.23	154.80 ± 21.55			
Gender of children						
Male	339	53.47	154.25 ± 24.98	−0.184	0.854	0.015
Female	295	46.53	154.61 ± 24.13			
Only child						
Yes	368	58.04	154.34 ± 25.23	−0.099	0.922	0.008
No	266	41.96	154.53 ± 23.67			
Marital status of parents						
Married	622	98.11	154.63 ± 24.08	0.865	0.405	0.310
Divorced/Widowed/Remarried	12	1.89	143.75 ± 43.42			
Education						
Junior school and below	73	11.51	142.92 ± 29.98	14.78	<0.001 *	0.045
Senior high school/Vocational school	316	49.84	152.99 ± 25.11			
College and above	245	38.64	159.69 ± 20.42			
Family composition						
Nuclear/Extended family ^b^	628	99.05	154.79 ± 24.12	2.30	0.069	1.148
Single-parent family/Other	6	0.95	116.10 ± 41.09			

^a^ Effect size: we calculated Cohen’s *d* for independent t-tests and partial eta squared ($\eta_{p}^{2}$ ) for ANOVAs to estimate the effect size. Effect sizes are indicated as small (*d* = 0.2, $\eta_{p}^{2}$ = 0.01), medium (*d* = 0.5, $\eta_{p}^{2}$ = 0.06), and large (*d* = 0.8, $\eta_{p}^{2}$ = 0.14) effects by Cohen’s *d* and $\eta_{p}^{2}$. ^b^ a nuclear family refers to a family consisting of parents and children; an extended family refers to a family consisting of three or more generations. * *p*-value < 0.05.

**Table 2:** Reliability of CPHLQ-Physical Part.

Dimension	Questions	Cronbach’s α	Split-Half Spearman-Brown
Gen-HL	39	0.915	0.889
HC-HL	12	0.831	0.722
DP-HL	16	0.874	0.847
HP-HL	11	0.619	0.650

Gen-HL, General Health Literacy; HC-HL, Health Care-Health Literacy; DP-HL, Disease Prevention-Health Literacy; HP-HL, Health Promotion-Health Literacy.

**Table 3:** Reliability of CPHLQ-Psychological Part.

Dimension	Questions	Cronbach’s α	Split-Half Spearman-Brown
Gen-HL	35	0.918	0.870
HC-HL	10	0.772	0.718
DP-HL	17	0.856	0.840
HP-HL	8	0.807	0.761

Gen-HL, General Health Literacy; HC-HL, Health Care-Health Literacy; DP-HL, Disease Prevention-Health Literacy; HP-HL, Health Promotion-Health Literacy.

**Table 4:** Construct validity of CPHLQ-Physical Part.

Model	χ^2^/df	RMSEA	GFI	CFI	IFI	PNFI
HC-HL	4.846	0.078	0.949	0.930	0.930	0.595
DP-HL	4.808	0.078	0.911	0.920	0.921	0.707
HP-HL	4.002	0.069	0.959	0.909	0.910	0.595

HC-HL, Health Care-Health Literacy; DP-HL, Disease Prevention-Health Literacy; HP-HL, Health Promotion-Health Literacy; χ^2^/df, relative chi-square; RMSEA, root mean square error of approximation; GFI, goodness-of-fit index; CFI, comparative fit index; IFI, incremental fit index; PNFI, parsimonious normed fit index.

**Table 5:** Construct validity of CPHLQ-Psychological Part.

Model	χ^2^/df	RMSEA	GFI	CFI	IFI	PNFI
HC-HL	4.936	0.079	0.954	0.933	0.933	0.591
DP-HL	4.443	0.074	0.920	0.900	0.901	0.708
HP-HL	3.120	0.058	0.981	0.979	0.979	0.520

HC-HL, Health Care-Health Literacy; DP-HL, Disease Prevention-Health Literacy; HP-HL, Health Promotion-Health Literacy; χ^2^/df, relative chi-square; RMSEA, root mean square error of approximation; GFI, goodness-of-fit index; CFI, comparative fit index; IFI, incremental fit index; PNFI, parsimonious normed fit index.

**Table 6:** Floor and ceiling effect of CPHLQ.

	Dimension	Questions	Floor Effect	Ceiling Effect
CPHLQ- Physical Part	Gen-HL	39	0.00%	0.00%
	HC-HL	12	0.01%	0.00%
	DP-HL	16	0.00%	0.01%
	HP-HL	11	0.00%	0.01%
CPHLQ- Psychological Part	Gen-HL	35	0.00%	0.00%
	HC-HL	10	0.00%	0.01%
	DP-HL	17	0.00%	0.00%
	HP-HL	8	0.01%	0.01%

Gen-HL, General Health Literacy; HC-HL, Health Care-Health Literacy; DP-HL, Disease Prevention-Health Literacy; HP-HL, Health Promotion-Health Literacy.

**Table 7:** Multiple linear regression of parental health literacy.

Characteristic	β	95% CI		*p*	VIF
		Lower	Upper		
Respondent					
Father/other	Reference	Reference		Reference	Reference
Mother	0.152	0.076	0.228	<0.001 *	1.022
Permanent residence registration					
Non-local	Reference	Reference		Reference	Reference
Local	0.019	−0.057	0.094	0.624	1.016
Age of children					
0–1 year	Reference	Reference		Reference	Reference
1–2 years	0.030	−0.054	0.115	0.480	1.270
2–3 years	0.046	−0.039	0.131	0.288	1.282
Gender of children					
Female	Reference	Reference		Reference	Reference
Male	0.005	−0.071	0.080	0.900	1.013
Only child					
No	Reference	Reference		Reference	Reference
Yes	−0.023	−0.099	0.054	0.557	1.042
Marital status of parents					
Divorced/widowed/remarried	Reference	Reference		Reference	Reference
Married	0.008	−0.072	0.089	0.836	1.148
Education					
Junior school and below	Reference	Reference		Reference	Reference
Senior high school/vocational school	0.201	0.077	0.325	0.002 *	2.737
College and above	0.322	0.197	0.446	<0.001 *	2.774
Family composition					
Single-parent family/other	Reference	Reference		Reference	Reference
Nuclear/extended family ^a^	0.128	0.047	0.209	0.002 *	1.169

^a^ Nuclear family refers to a family consisting of parents and children; an extended family refers to a family consisting of three or more generations. * *p*-value < 0.05.

## Data Availability

The datasets used and analyzed during the current study are available from the corresponding author upon reasonable request.
